# Assessments of a novel digital follow-up tool Rehabkompassen^®^ to identify rehabilitation needs among stroke patients in an outpatient setting

**DOI:** 10.1177/20552076221104662

**Published:** 2022-06-03

**Authors:** Xiaolei Hu, Karolina Jonzén, Marcus Karlsson, Olof A Lindahl

**Affiliations:** 1 Department of Community Medicine and Rehabilitation, 8075 Umeå University, Umeå, Sweden; 2 Department of Radiation Sciences, Radiation Physics, Biomedical Engineering, 8075 Umeå University, Umeå, Sweden

**Keywords:** Stroke rehabilitation, need assessment, eHealth, digital tool, usability, feasibility, outcome assessment, follow-up, outpatient setting

## Abstract

**Introduction:**

It remains a huge challenge to identify individual rehabilitation needs in a time-efficient manner for providing patient-tailored rehabilitation during the continuum of stroke care. We have recently demonstrated the usefulness of a paper-version Rehab-Compass as a follow-up tool. The aim of the current study was to develop a digital version of the Rehab-Compass and evaluate its usability and feasibility.

**Methods:**

The novel digital tool Rehabkompassen^®^ was developed by an iterative and participatory design process. Patients’ rehabilitation needs were visualized by the tool and used before, during, and after the consultation. The usability and feasibility of the tool was assessed by task completion rate, the System Usability Scale, and satisfaction questionnaires among 2 physicians and 24 adult stroke patients in an outpatient clinical setting.

**Results:**

Rehabkompassen^®^ identified and graphically visualized a panoramic view of the stroke patients’ multidimensional needs in individual- and group levels. The instrument appeared to be feasible and time efficient in clinical use with a 100% overall task completion rate for both patients and physicians. A majority of the patients reported that it was very easy or fairly easy to answer the digital questionnaires and to understand their own digital Rehab-Compass graph. Two physicians reported a high mean score on the System Usability Scale (95/100) and were positive about using the tool in the future.

**Conclusions:**

The current results indicated that Rehabkompassen^®^ was a feasible, useful, and time-saving follow-up tool for the identification of rehabilitation needs among stroke survivors in the post-acute continuum of care after stroke. Further research is needed to evaluate the efficacy of the digital instrument among stroke patients.

## Introduction

Stroke is a leading cause of disability with various long-term impairments and restrictions in social participation and quality of life among adults worldwide.^1–3^ A recent Global Burden of Disease study reported 12.2 million incident cases of stroke with 101 million prevalent cases of stroke and 143 million disability-adjusted life years due to stroke globally.^3^ Thus, there is an urgent need to better identification of individual rehabilitation needs for providing patient-tailored rehabilitation in a time-efficient way to reduce disability among persons with stroke and diminish the socioeconomic burden.

To meet these challenges, we have created and developed a follow-up tool that illustrates the patient's reported health status graphically in a paper-version “Rehab-Compass”.^4^ The tool is similar to the Post Soft Care-App,^5^ where the Post Stroke Checklist (PSC) is used to highlight the rehabilitation needs among persons with stroke. However, the Rehab-Compass has been considered easier to capture subtle dynamic profiles of stroke impact during long-term follow-ups compared to the dichotomous construction of PSC.^4,6^ This paper-version Rehab-Compass has been proved to be a feasible, useful, and time-saving tool for identification of unmet rehabilitation needs over time among stroke- and transient ischemic attack (TIA) survivors.^4,7^ However, the data collection is paper based and the Rehab-Compass graphs have to be manually generated in Microsoft Excel, which has been a time-consuming burden for the medical staff. Together with many other potential benefits of digitalization in health care,^8^ such as improvements in the accessibility and quality of care with significant cost savings, there is an urgent need to digitalize the whole process from data collection to generation and presentation of results in order to make the instrument more efficient in clinical practice.

Moreover, conducting usability and feasibility assessments on digital applications, with regard to both patients and healthcare professionals in an outpatient setting, are considered one of the key requirements within eHealth technology.^9^ However, the number of usability studies on digital health applications has not increased at an equivalent rate, despite the diversity of usability assessment methods and the exponential growth of eHealth applications.^10^ Since digital applications are often challenging to fit onto health problems and healthcare systems,^11,12^ it's important to conduct usability and feasibility evaluations on newly developed digital applications among both patients and healthcare professionals. The usability and feasibility assessments can ensure that the end users’ needs in the healthcare system are appropriately targeted in order to improve accessibility and reduce any potential risks.

The aims of this study were to develop a digital tool, Rehabkompassen^®^, based upon the paper-version Rehab-Compass;^4^ and then preliminary evaluate the usability and feasibility of the newly developed instrument among both stroke patients and health care professionals in an outpatient clinical setting.

## Materials and methods

### Instrument design and development

**Figure 1.**
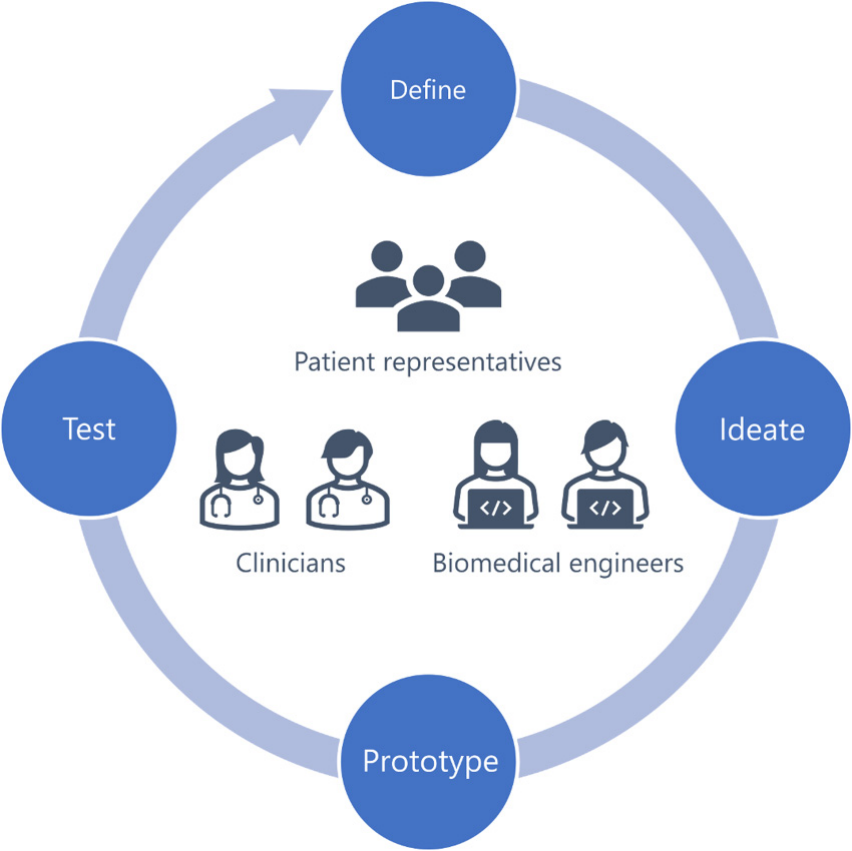
Iteration cycle during the development process, incorporating both clinical and technical experts as well as patient representatives.

Rehabkompassen^®^ was developed in-house as a Windows application. The concept of the tool was to identify and graphically visualize a panoramic view of stroke patients’ heterogenic rehabilitation needs based on 6 well-validated and reliable patient-reported outcome measurements (PROMs), as demonstrated previously in the paper-version (Figure 1(a)).^4^ The PROMs used in Rehabkompassen^®^ were Stroke Impact Scale (SIS) version 3.0 with additional questions related to sensory disturbances, sleep disturbances, and natural topics; Hospital Anxiety and Depression Scale (HAD); Fatigue Assessment Scale (FAS); the simplified modified Rankin Scale questionnaire (smRSq) and EuroQoL 5-dimension 3 levels (EQ-5D-3L) as well as Eating Assessment Tool (EAT-10) added for evaluating swallow function. In order to easily describe these questionnaires, we named them as Rehabkompassen^®^ questionnaires even though they were the existing questionnaires used in the instrument.

The paper-based Rehab-Compass^4^ was further digitalized during 2016–2020 using a participatory iterative design approach where various stakeholders are engaged in the design process to increase user value (Figure 1).^13^

**Figure 2.**
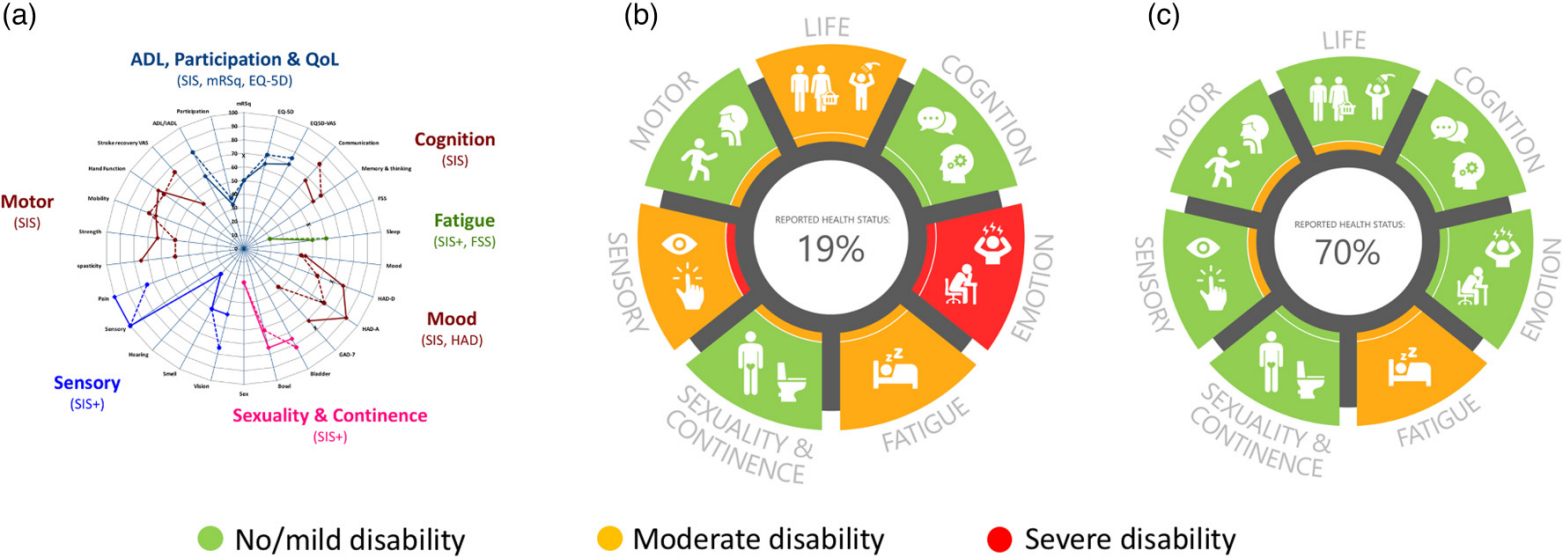
Rehabkompassen^®^ developed from the original paper-version^4^ (a) to the digital version that identified more (b) or less (c) unmet rehabilitation needs among persons after stroke onset.

Both stroke patients and multi-professional medical staff with expertise in stroke rehabilitation worked closely with experts in biomedical engineering and human interaction design during the development of the instrument. A series of prototypes were iteratively built and tested with patient representatives and health care professionals using small-scale qualitative interviews and user testing with think-aloud protocol to reach a final prototype (Figure 2(b) to (c)).

### Assessing clinical usability of the Rehabkompassen^®^

*Study design*: The evaluation on the usability and feasibility of the tool was conducted in a cohort study at the the Department of Neurological Rehabilitation in close collaboration with the Department of Biomedical Engineering – Research and Development in the University Hospital of Umeå, Sweden.

A total of 100 patients diagnosed with a stroke at Stroke Center, University Hospital of Umeå during November 2020–March 2021 were assessed for study eligibility. Inclusion criteria were both males and females aged > 18 years who suffered a stroke at least 3 months before a visit to the outpatient clinic. The participants needed to have been discharged from the hospital and live in the community when they participated in the study. Exclusion criteria were the inability to answer the evaluation questions or to see the Rehab-Compass graph. In the end, 24 of 100 persons after stroke participated in the study using Rehabkompassen^®^ as a follow-up tool at the 12-month follow-up with written consent. A total of 15 among 100 patients declined due to technical hinder (n = 11) or didn’t meet the selection criteria (n = 4). Meanwhile beside 60 patients were not interested to participate in the study, and one died.

*Digital process from the questionnaires to the Rehab-Compass Graph by Rehabkompassen^®^*: * *One month before the follow-up, a research nurse sent out questionnaires to the participants through 1177.se which is the Swedish government-issued digital platform for citizens’ healthcare. Patient participants answered the digital questionnaires regarding their health via 1177.se at home, latest 1 week before the follow-up. A research nurse assisted participants when needed, by providing guidance or help over the phone or in person.

**Figure 3.**
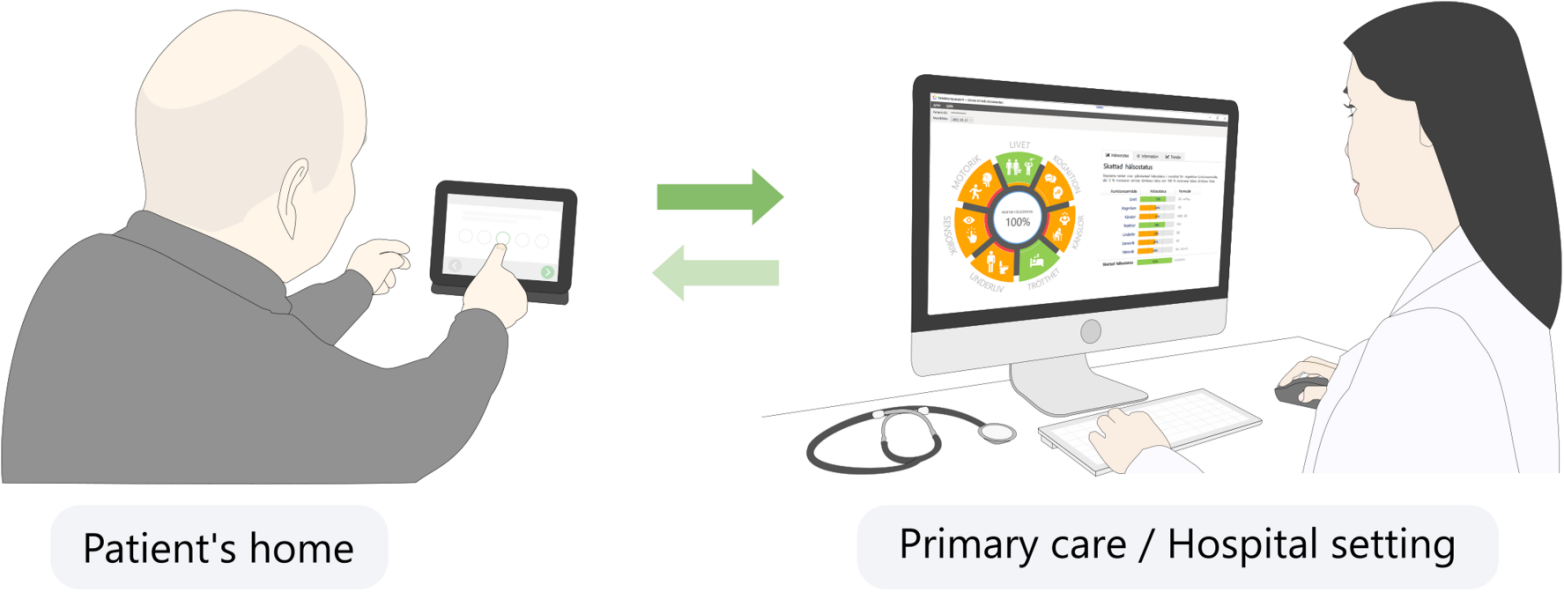
Utilization of Rehabkompassen^®^ in the health care system where a person with stroke fills the digital questionnaires at home prior to an outpatient visit and the medical professionals can use patient's Rehab-Compass graph before, during, and after the outpatient visit.

After completion of questionnaire answering, the results were exported by a research nurse to a secure server at the clinic; and thereafter automatically transformed into a digital Rehab-Compass graph, viewable to the physician or other medical staff at the clinic via the Rehabkompassen^®^ tool on a computer (Figure 3).

*Utilization of Rehabkompassen^®^ in the continuum of care after stroke*: After the patient filled out the digital questionnaires at home and the Rehab-Compass graph was generated, the healthcare practitioner was able to use the tool as a support for initial triage or plan on the needs of patients’ rehabilitation and staff resource before the visit.

During the outpatient visit, the physician presented the patient's Rehab-Compass graph and discussed the rehabilitation needs with the patient in detail, which would potentially rule out or adjust the eventual over- or underestimation of their functioning in the PROMs. Together with medical information and examination during the visit, the instrument provided patients’ perceived health status to the physician/clinical practitioner to advice and help them prioritize different interventions. This would facilitate a patient-tailored rehabilitation. Thus, the physiatrist/ stroke physician used Rehabkompassen^®^ as an assistance tool for shared decision making on the patients’ rehabilitation needs during the outpatient visit.

In cases where the patient had multidimensional problems, they would be referred to a rehabilitation team for providing different rehabilitation interventions when needed. The patient's own Rehab-Compass graph was used in the rehabilitation team consisting of different professionals in the clinic. Together with the patient, the rehabilitation team finalized a formal rehabilitation plan addressing usually two to three main issues that needed to be treated under one rehabilitation period. In another case where a patient needed to be referred to another clinic, the patient's own Rehab-Compass graph was attached with the referral to clarify the patient's rehabilitation needs. In addition, this tool enabled the rehabilitation team and the patient toassess the alterations of rehabilitation needs over time. For example, two Rehab-Compass graphs generated at 3- and 12-month follow-ups respectively were compared, simply by comparing the color changes in the different functioning domains, (see examples in Figure 2(b) and (c)).

*Multiple assessments on usability and feasibility of Rehabkompassen^®^*: The usability and feasibility of the tool were evaluated by how many participants (patients and physicians) completed the study protocol.

**Figure 4.**
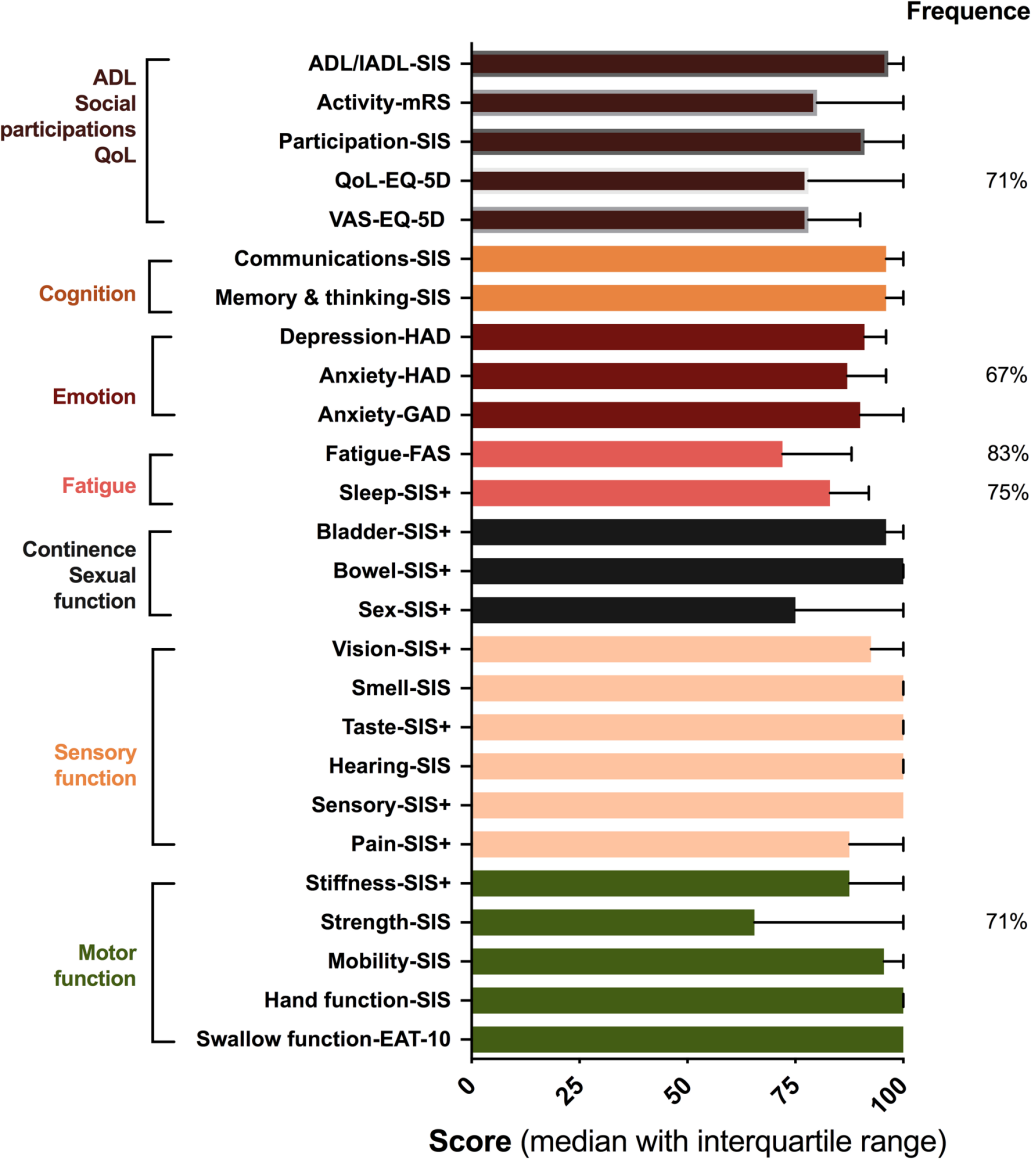
Patients’ overall experiences (a-c) on using Rehabkompassen^®^ during the outpatient visit.

After the visit at a 12-month follow-up, the patient participants answered a satisfaction questionnaire through 1177.se. The patient satisfaction questionnaire consisted of their technical background, their experience of filling the digital questionnaires, and their satisfaction with Rehabkompassen^®^ during the outpatient visit. The various degrees of satisfaction were rated in terms of how easy it was to understand the Rehab-Compass graph and how it affected their ability to understand their rehabilitation needs during the consultation. Each question was answered using a Likert scale, ranging from 1 to 5 on two questions and ranging from 1 to 3 on one question (Figure 4). Additionally, the questions were followed by subsequent open-ended questions to allow respondents to motivate their answers.

To assess the general perceived usability of Rehabkompassen^®^ among medical staff, the two physicians involved in the study answered the System Usability Scale (SUS)^14^ questionnaire. The SUS is a widely adopted and validated instrument with 10-item Likert scale questions with five response options from strongly agree to strongly disagree. The total score is ranged from 0 to 100 with higher scores indicating better usability.

### Data presentation and statistics

The data from the Rehabkompassen^®^ questionnaires were converted into a 0–100 scale but unchanged in terms of variable properties, where 100 represented the best condition and 0 represented the worst^4^ To further facilitate interpretation of the results, both functional areas and bar charts were color-coded on a general scale from worst possible health (0–30) in red to best possible health (70–100) in green and values in between in orange, besides using already existing cut-offs for individual PROMs (Figure 2(b) and (c)).^15^^–^^18^ The colors of the functional areas represented the mean value of the included domains.

Mean ± SD, number, and % of case were used to present the samples’ characterization and the satisfaction questionnaires when appropriate. Group data on the outcome measurements are presented as median with IQR (25%–75% percentile). The statistical analyses were performed using the software GraphPad Prism, version 9.0 (San Diego, CA, USA) or Microsoft Excel when appropriate.

## Results

### Development and refinement of the instrument

During the iterative and participatory design process (Figure 1), we collected different ideas and concepts from the research team based on the scientific paper-based Rehab-Compass graph (Figure 2(a)). A nonfunctional prototype was developed and tested with two patient representatives and two health care professionals in each iterative circle. A total of five iterative circles were carried out. Based on user feedback received during the iterative development process, an extra color-coded field, representing the lowest function value, was added to the inner edge of each area within the Rehab-Compass graph (Figure 2(b) and (c)), to avoid the risk of the clinician missing individual disturbed functions.

The refined digital Rehab-Compass graph, namely Rehabkompassen^®^ (Figure 2(b) and (c)), presented stroke patients’ self-reported health status in a holistic view with seven areas commonly affected after stroke: life, cognition, emotion, fatigue, sexuality and continence, sensory function, and motor function. Each area consisted of several domains, presented individually in bar charts to the side of the Rehab-Compass graph when selecting the associated area (see the charts on the computer screen in Figure 3).

### Participant recruitment and characteristics

A total of 24 individuals after stroke, aged between 42 and 86 (mean age = 68) with equal female to male representation, participated in the study (see characteristics in Table 1)*.* More than half of the participants (13/24) had university degrees and 22/24 rated their computer skills as average or good, while only two participants identified themselves as beginners. A majority had previous experience with 1177.se, with only two having never logged into the platform before participating in this study even though one of them was considered with the average computer skills. Computer (12/24) and mobile phone (7/24) were the dominant devices used to answer the questionnaires. Even though participants were advised by the research nurse to fill in the questionnaires on different days to avoid tiredness, most of them (15/24) completed all questionnaires at once. A majority (21/24) were also able to answer the questionnaires without any assistance. Three participants required some or extensive assistances provided by their proxies under guidance of the research nurse.

**Table 1.** Baseline participant characteristics.

Characteristic	Categories	Total (24) n (%)
Age	Mean (SD)	68.25 (11.6)
	Median	71.5
Sex	Female	12 (50.0)
	Male	12 (50.0)
Highest completed education	No completed education	1 (4.2)
	Primary school or equivalent	2 (8.3)
	High school or equivalent	8 (33.3)
	Postsecondary education, university or college	13 (54.2)
Computer skills	Beginner	2 (8.3)
	Average	12 (50.0)
	Good	10 (41.7)
	Expert	0 (0.0)
No. of times previously logged in to 1177's Health Guide E-Services	0	2 (8.3)
	1–5	2 (8.3)
	> 5	20 (83.3)
Device used to answer questionnaires	Computer	12 (50.0)
	Tablet	0 (0.0)
	Mobile phone	7 (29.2)
	Different devices	5 (20.8)
Level of assistance	No assistance	21 (87.5)
	Some assistance	2 (8.3)
	Extensive assistance	1 (4.2)
Answered questionnaires at different occasions	Yes	9 (37.5)
	No	15 (62.5)

n is equal to the number and % is equal to the percentage of the total number.

### Identification of rehabilitation needs on an individual level

The digital instrument presented an overview of the individual patient's health status to facilitate the determination of various rehabilitation needs among different patients (Figure 2(b) and (c)). One patient presented moderate to severe impairments (Figure 2(b)) not only in emotion and pain but also in fatigue aspects, which resulted in moderate activity limitation and severe participation restrictions. In contrast, another patient (Figure 2(c)) had only mild problems with sleep disturbance and fatigue, which led to certain participation restrictions, even though the patient had no limitation at all in daily activity. When comparing patients 2B and 2C via their Rehab-Compass graphs, more rehabilitation needs were observed in patient 2B than patient 2C. Figure 2(b) and (c) could also be an example of the same patient at 3- and 12-month follow-ups, indicating the alteration of rehabilitation needs over the time.

### Identification of rehabilitation needs on a group level

**Figure 5.**
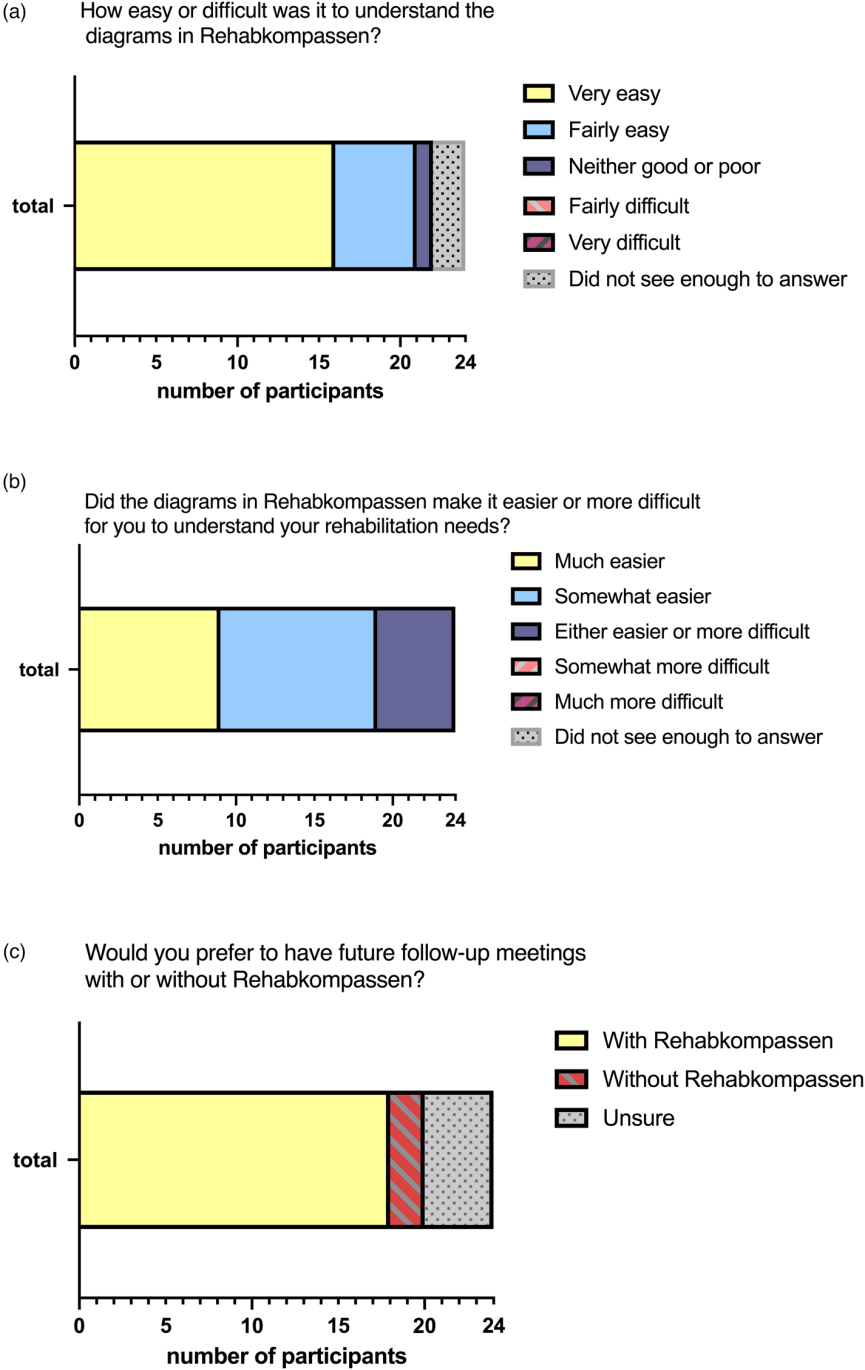
Extents and frequency of unmet rehabilitation needs in persons with stroke, identified by the Rehabkompassen^®^ at 12-month follow-ups. The different conditions were assessed by various instruments and grouped into different domains on the left. A value <100 in the individual domain in Stroke Impact Scale (SIS) and SIS + was defined as impairment. The bar graph represents extents of unmet rehabilitation needs with 0 (worst outcome/unmet need) to 100 (best outcome/ no need). Numbers on the right side of the graph represent some of the frequencies of various conditions.

On a group level, the tool identified a wide range of stroke-related problems among 24 participants at the 12-month follow-up (Figure 5). The most severe problems reported in median (25%–75% percentile) by the cohort were strength (65 (50–100)), fatigue (72 (50–91)), and sexual dysfunction (75(50–100)), followed by quality of life (QoL) (78 (63–100)) and activity (80 (80–100)). In terms of frequency, fatigue (20/24, 83%) and sleep disturbances (18/24, 75%) were the most commonly reported problems followed by QoL (17/24, 71%), strength (17/24, 71%) as well as anxiety (16/24, 67%).

### Usability and feasibility assessment

All 24 patient participants and 2 physicians completed the whole procedure, which gave a 100% completion rate and indicated high usability and feasibility of the tool. There was no serious or critical issue reported from the patient participants or the physicians.

### Satisfaction of answering the digital questionnaires

Overall, the participants were satisfied with answering the digital questionnaires, with 22 of 24 participants rating it as very easy or fairly easy. Furthermore, a majority did not feel like the questionnaires were demanding to answer even though there were a total of 130 questions used in the instrument.

### Usability and satisfaction of the rehabkompassen^®^ tool among patients

At the 12-month follow-up, 21 of 24 patient participants reported a very good or fairly good understanding of their Rehab-Compass graph (Figure 4(a)). Meanwhile, two participants did not feel like they had seen enough to answer the question; some of them didn’t remember what the digital Rehab-Compass was. A majority (19/24) reported the graph facilitated understanding of their rehabilitation needs (Figure 4(b)). Most of the participants (18/24) considered to use the instrument in the future (Figure 4(c)).

### General usability and satisfaction of the Rehabkompassen^®^ tool among physicians

**Figure 6.**
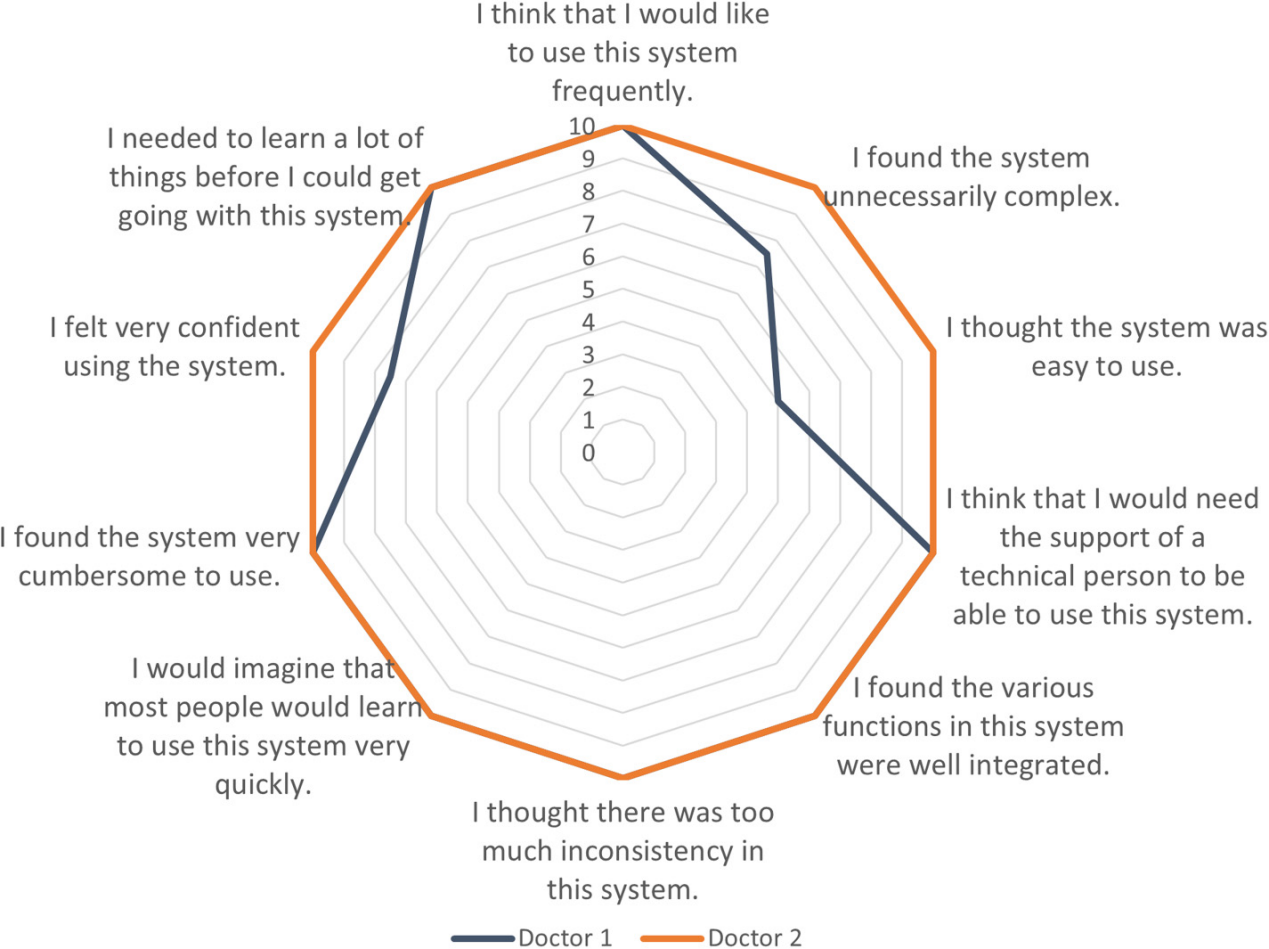
Two physicians’ general perceived usability of the Rehabkompassen^®^ tool using the System Usability Scale (SUS).

Two physicians, one male and one female aged between 40 and 52, respectively, used the Rehabkompassen^®^ tool when they met the patient participants during the outpatient visits. Overall, both rated a very high usability with a mean SUS score of 95 (Figure 6). Both physicians considered the tool having well-integrated functions without too much inconsistency. They reported the tool was quick to learn and not cumbersome to use. One physician rated lower on the ease of use and suggested integration of more detailed information into the software. Both felt confident in using the tool; and were positive about using it for future outpatient visits.

## Discussion

In this study, we developed and refined the paper-version Rehab-Compass graph based on six well-validated PROMs^4^ into a novel digital follow-up tool, namely Rehabkompassen^®^. With a graphical visualization, the digital Rehabkompassen^®^ provided a user-friendly panoramic view of the multidimensional needs on both individual and group levels. Based on its PROMs nature, the tool can be used as a screening/triage tool before the outpatient visit, a communication tool during the outpatient visit, and a long-term follow-up tool in a patient-centered manner. Both patients and doctors had a 100% task completion rate in the current study, which indicated high usability and feasibility of the tool for the healthcare professionals and persons with stroke during the outpatient clinic visits. A majority of the patients reported that it was very easy or fairly easy to understand their own Rehab-Compass graph and that it facilitated understanding of their rehabilitation needs. Two doctors rated a high mean SUS score at 95/100. Both patients and physicians were positive to use the instrument in the future.

Rehabkompassen^®^ delivers a new way of displaying PROM data with a panoramic overview of individual unmet rehabilitation needs according to the concept of International Classification of Functioning (ICF), disability, and health.^19^ One major advantage of the instrument is the presentation of multidimensional needs in the same graph, covering not only several functional domains but also information on activities of daily living (ADLs) and instrumental ADL, participation, and quality of life (under the functional area “Life” in the Rehab-Compass graph). Thus, the tool may make it easier to highlight various activity limitations and its’ related function impairments as well as hidden issues, such as depression, anxiety, and/or fatigue. In this way, Rehabkompassen^®^ may also promote patient-tailored rehabilitations. Based on the feedback from both patients and physicians, the instrument facilitated the quick capture of a patient's actual unmet rehabilitation needs in clinical practice, as previously demonstrated in the paper version of the Rehab-Compass graph.^4^ Additionally, the digitalized process saved time and cost for the healthcare practitioners who did not have to manually input the collected data as in the original paper-version Rehab-Compass.^4^

Using the Rehabkompassen^®^ tool, we were able to enhance the patient-centered care in several steps around an outpatient visit since the instrument was based on PROMs.^20,21^ For example, the instrument was used as a screening tool for initial triage on the needs of patients’ rehabilitation and staff resources *before* the visit. Meanwhile, the instrument provided a color-coded communication platform between the patient and healthcare professionals *under* the visit and/or during the patient's transfer between different care levels *after* the visit. Since the severity of patients’ problems/ rehabilitation needs is illustrated with different colors by the tool, this may facilitate the healthcare professionals to capture the patients’ needs during the consultation. The discussion with the patient and physical examination during the consultation will further adjust the eventual over- or underestimation of patient's functioning from the answers of the PROMs. Together with the tool, a shared decision would thereafter be generated to facilitate more patient-tailored rehabilitation interventions. In addition, the Rehabkompassen^®^ questionnaires can be filled again when needed. It can thereby be used as a follow-up tool *after* the eventual rehabilitation regimens have been delivered. The alterations of rehabilitation needs overthe time were illustrated by comparing two different Rehabkompassen graphs at different time-points (e.g. see Figure 2(b) and (c)). Our results indicated the broad usefulness of the tool to facilitate a patient-tailored rehabilitation in the continuum of post-acute care after stroke.

Very high usability and feasibility of the Rehabkompassen^®^ tool were demonstrated by both patients and physicians in the study. This suggests that the end users’ needs were appropriately targeted in the clinical practice since the instrument was iteratively refined based on feedback from the end users.^12^ This enhanced feasibility and usability of the instrument is supported by previous findings that a user-centered, interdisciplinary and collaborative approach to mHealth innovations enhances feasibility, acceptability, and usability in the healthcare system.^22^ Notably, 15 of 100 patients declined to participate in the study due to either the technical issues or the severe impairments. This indicates that extra support and help to these vulnerable individuals are definitively required in order to provide equal care to the whole stroke population.

The patient participants in this study were generally positive about answering the digital questionnaires, although they were quite extensive with about 130 questions in total. It's possibly due to the flexibility offered by the digital instrument, allowing patients to answer the questionnaires whenever they want to. The current data was somewhat contradictory to some studies^23^^–^^25^ that have pointed out the challenge of using electronic PRO systems in elderly patient groups. The reason for this discrepancy could partly be explained by the generally high education level and computer skills in this cohort. However, this may not truly represent the whole stroke population dominant with the elderly without sufficient computer knowledge. Furthermore, leveraging the patients’ digital healthcare platform 1177.se may also have contributed to the positive results since a majority had previous experience with the platform. Still, some individuals declined to participate in the study due to not being able to log in to 1177.se or having no computer experience. Hence, extra service should be provided to these vulnerable individuals after stroke in order to assist them in answering the digital questionnaires before the outpatient visit. This will be a crucial step to ensure an equal quality of care in the near future, regardless of computer skills and physical and cognitive ability.

Most of the patient participants reported that the visualization of their own Rehab-Compass graph was easy to understand and helpful in identifying their health issues and rehabilitation needs since the patients’ own experienced needs were often not so clear for themselves. This is consistent with the previous findings.^45^ Intriguingly, even though 21 patients reported that their Rehabkompassen graphs were easy to understand but only 18 patients were willing to use the tool in the future. The reason for this discrepancy is unknown, but it's of interest to go through the open-ended feedbacks to find a possible answer in the future.

In the study, a very high SUS score (95/100) was reported by the two physicians who used the Rehabkompassen^®^ tool during the outpatient visits. Together with 100% task completion rate, our results indicated higher usability and feasibility of the tool compared to other telerehabilitation portals and the mobile apps (respectively, SUS score at 78 and 71).^26^ As demonstrated in the SUS questionnaire, Rehabkompassen^®^ was considered a well-integrated tool with good consistency. It was also easy to learn and fairly easy to use as well as time saving, as demonstrated previously in clinical practice.^4,7^ This was further confirmed by both physicians being positive to use Rehabkompassen^®^ in the future.

The strength of the study was the use of multiple assessments of usability since the SUS is insufficient as a stand-alone usability benchmark for eHealth.^26^ The end users’ feedbacks were used to both improve the tool and to further ameliorate the planned future studies. Moreover, both patients and medical staff involved in the development and evaluation of the instrument provided extra strength to the study, since a majority of usability studies are performed by patients or medical staff only.^10^ Of course, we are aware of the small sample size and the need for further studies to confirm the efficacy of the instrument and include other professions in the rehabilitation team to evaluate the tool. Additionally, 3 of 24 patients needed their proxy to fill in the questionnaires, which have raised certain concerns about the eventual accuracy of the reported impairments presented by the tool. However, the discussion with the patient and physical examination during the consultation will help to diminish the risk of incorrect rehabilitation. Furthermore, integrating the instrument with the electronic health record system will be crucial in the near future to secure a seamless and safe clinical use.^27^^–^^29^

## Conclusions

In conclusion, the novel digital Rehabkompassen^®^ tool was developed, refined, and evaluated among stroke survivors and health care professionals in the outpatient setting. The tool seems to be feasible and useful for the identification of rehabilitation needs in a time-efficient manner with its’ multifunction as screening, communication, and follow-up tool in the post-acute continuum of care after stroke. This will further provide patient-tailored rehabilitation in the continuum of care after stroke and thereby save long-term suffering and cost for the society. Further research is needed though to evaluate the efficacy of the tool in large-scale study among stroke patients and various medical professions.

## References

[1] Kleindorfer DO Khatri P . Understanding the remarkable decline in stroke mortality in recent decades. Stroke 2013; 44: 949–950. Comment Editorial Research Support, N.I.H., Extramural 9 March 2013. 23471265 10.1161/STROKEAHA.111.000560

[2] Ovbiagele B Goldstein LB Higashida RT , et al. Forecasting the future of stroke in the United States: a policy statement from the American heart association and American stroke association. Stroke 2013; 44: 2361–2375. 23697546 10.1161/STR.0b013e31829734f2

[3] Collaborators GBDS. Global, regional, and national burden of stroke and its risk factors, 1990–2019: a systematic analysis for the global burden of disease study 2019. Lancet Neurol 2021; 20: 795–820. 34487721 10.1016/S1474-4422(21)00252-0 PMC8443449

[4] Magaard G Wester P Levi R , et al. Identifying unmet rehabilitation needs in patients after stroke with a graphic rehab-compass(TM). J Stroke Cerebrovasc Dis 2018; 27: 3224–3235. 30097401 10.1016/j.jstrokecerebrovasdis.2018.07.013

[5] De Bartolo D Morone G Lupo A , et al. From paper to informatics: the Post Soft Care-App, an easy-to-use and fast tool to help therapists identify unmet needs in stroke patients. Funct Neurol 2018; 33: 200–205. 30663966

[6] Ward AB Chen C Norrving B , et al. Evaluation of the post stroke checklist: a pilot study in the United Kingdom and Singapore. Int J Stroke 2014; 9: 76–84. 25088427 10.1111/ijs.12291

[7] Magaard G Stålnacke B Sörlin A , et al. Identifying rehabilitation needs among individuals after transient ischemic attack with a rehab-compass as a simple follow-up instrument in the out-patient clinic. Journal of Rehabilitation Medicine Clinical Communications 2019; 2: 1–8. Original report 14 October 2019.. 10.2340/20030711-1000018 PMC8008729 33884119

[8] Blix M Levay C . Digitalization and Health Care- a report to Swedish Government’s expert group on public economics. ESO’s publication series, 2018, p. https://eso.expertgrupp.se/wp-content/uploads/2019/2008/Digitalization-and-health-care-2018_2016-English-version.pdf.

[9] Standardisation(ISO) IOf. Ergonomics of Human-System Interaction-Part 11: Usability: Definition and Concepts. 2018.

[10] Maramba I Chatterjee A Newman C . Methods of usability testing in the development of eHealth applications: a scoping review. Int J Med Inform 2019; 126: 95–104. 31029270 10.1016/j.ijmedinf.2019.03.018

[11] Zapata BC Fernandez-Aleman JL Idri A , et al. Empirical studies on usability of mHealth apps: a systematic literature review. J Med Syst 2015; 39: 1. 25600193 10.1007/s10916-014-0182-2

[12] Greenhalgh T Wherton J Papoutsi C , et al. Beyond adoption: a new framework for theorizing and evaluating nonadoption, abandonment, and challenges to the scale-up, spread, and sustainability of health and care technologies. . J Med Internet Res 2017; 19: e367. 29092808 10.2196/jmir.8775 PMC5688245

[13] Van der Velden M Mörtberg C . Participatory design and design for values. Handbook of Ethics, Values, and Technological Design: Sources, Theory, Values and Application Domains 2015; S: 41–66.

[14] Brooke J . SUS-A quick and dirty usability scale. Usability Evaluation in Industry 1996; 189: 4–7.

[15] Guidetti S Ranner M Tham K , et al. A "client-centred activities of daily living" intervention for persons with stroke: one-year follow-up of a randomized controlled trial. J Rehabil Med 2015; 47: 605–611. 26121986 10.2340/16501977-1981

[16] Herrmann C . International experiences with the hospital anxiety and depression scale--a review of validation data and clinical results. J Psychosom Res 1997; 42: 17–41. 9055211 10.1016/s0022-3999(96)00216-4

[17] Burton LJ Tyson S . Screening for mood disorders after stroke: a systematic review of psychometric properties and clinical utility. Psychol Med 2015; 45: 29–49. 25066635 10.1017/S0033291714000336

[18] Vellone E Savini S Fida R , et al. Psychometric evaluation of the stroke impact scale 3.0. J Cardiovasc Nurs 2015; 30: 229–241. 24695074 10.1097/JCN.0000000000000145

[19] Stucki G Cieza A Melvin J . The international classification of functioning, disability and health (ICF): a unifying model for the conceptual description of the rehabilitation strategy. J Rehabil Med 2007; 39: 279–285. 17468799 10.2340/16501977-0041

[20] Ginsburg GS Phillips KA . Precision medicine: from science to value. Health Aff (Millwood) 2018; 37: 694–701. 29733705 10.1377/hlthaff.2017.1624 PMC5989714

[21] Hudon C Fortin M Haggerty JL , et al. Measuring patients’ perceptions of patient-centered care: a systematic review of tools for family medicine. Ann Fam Med 2011; 9: 155–164. 21403143 10.1370/afm.1226 PMC3056864

[22] Matthew-Maich N Harris L Ploeg J , et al. Designing, implementing, and evaluating mobile health technologies for managing chronic conditions in older adults: a scoping review. JMIR Mhealth Uhealth 2016; 4:e5127. 10.2196/mhealth.5127 PMC4919548 27282195

[23] Hess R Santucci A McTigue K , et al. Patient difficulty using tablet computers to screen in primary care. J Gen Intern Med 2008; 23: 476–480. 18373148 10.1007/s11606-007-0500-1 PMC2359506

[24] Kavalieratos D Sullivan SM Hess R . Engaging cardiology patients via electronic patient-reported outcomes: a usability assessment. Am Heart Assoc 2014; 7: A213.

[25] Graf J Simoes E Wißlicen K , et al. Willingness of patients with breast cancer in the adjuvant and metastatic setting to use electronic surveys (ePRO) depends on sociodemographic factors, health-related quality of life, disease status and computer skills. Geburtshilfe Frauenheilkd 2016; 76: 535–541. 27239062 10.1055/s-0042-105872 PMC4873300

[26] Broekhuis M van Velsen L Hermens H . Assessing usability of eHealth technology: a comparison of usability benchmarking instruments. Int J Med Inform 2019; 128: 24–31. 31160008 10.1016/j.ijmedinf.2019.05.001

[27] Gibbons E Black N Fallowfield L , et al. Patient-reported outcome measures and the evaluation of services. *Challenges, solutions and future directions in the evaluation of service innovations in health care and public health*. NIHR Journals Library, 2016. 27227187

[28] Van Der Wees PJ Nijhuis–Van Der Sanden MW Ayanian JZ , et al. Integrating the use of patient–reported outcomes for both clinical practice and performance measurement: views of experts from 3 countries. Milbank Q 2014; 92: 754–775. 25492603 10.1111/1468-0009.12091 PMC4266175

[29] Holch P Warrington L Bamforth L , et al. Development of an integrated electronic platform for patient self-report and management of adverse events during cancer treatment. Ann Oncol 2017; 28: 2305–2311. 28911065 10.1093/annonc/mdx317 PMC5834137

